# Some practical issues about HLA-A29 in birdshot retinochoroiditis

**DOI:** 10.1186/s12348-023-00326-5

**Published:** 2023-03-09

**Authors:** Ioannis Papasavvas, Jonas J. W. Kuiper, Carl P. Herbort Jr

**Affiliations:** 1 Inflammatory and Retinal Eye Diseases, Centre for Ophthalmic Specialised Care (COS), Rue Charles-Monnard, 6, 1003, Lausanne, Switzerland; 2 Department of Ophthalmology, Center for Translational Immunology, University Medical Center Utrecht, Utrecht University, Utrecht, The Netherlands

**To the editor**

HLA-A29 birdshot retinochoroiditis (BRC) is a bilateral posterior uveitis which affects simultaneously, although independently, the retina and the choroid. The retinal presentation is marked by vasculitis, involving both small and large vessels, papillitis and macular oedema while choroidal involvement takes the form of a primary stromal choroiditis, with active, often occult, foci occupying the middle stroma of the choroid only detected by indocyanine green angiography (ICGA) at onset. These foci give rise, after several months of evolution without treatment, to cicatricial areas, appearing as oval cream-coloured lesions of the posterior pole and mid periphery that do not appear any more on ICGA as they are not full-thickness scars [1] (Figs. 1 and 2). BRC It is classified as an MHC-I (major histocompatibility complex class I)-opathy [2, 3], with the MHC-I antigen HLA-A29 occurring in ~ 100% of cases (most commonly the HLA-A*29:02 allele) compared to 5–10% of the Western population. In addition, MHC-I pathway genes *ERAP1* and *ERAP2* are genetically associated with BRC [3]. The combination of these genes contributes to a higher disease risk than each gene alone, indicating that ERAP1, ERAP2, and HLA-A29 work together in the pathogenesis of BRC [3], which challenges the view that BCR also occurs in HLA-A29-negative cases.

**Fig. 1:**
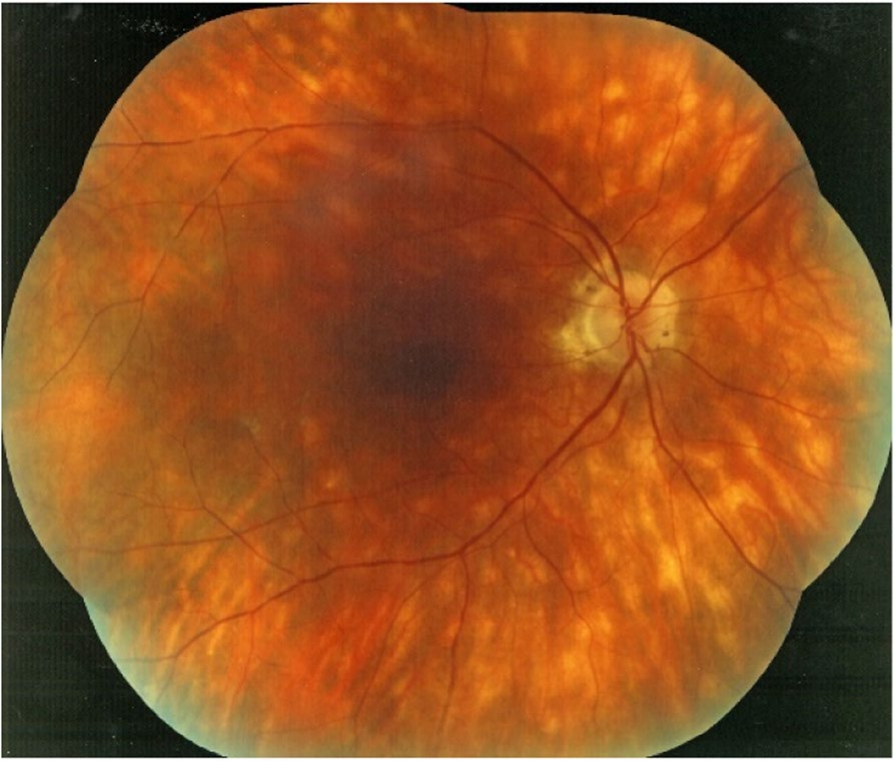
Advanced case of BRC with numerous cream-coloured BRC fundus lesions

**Fig. 2:**
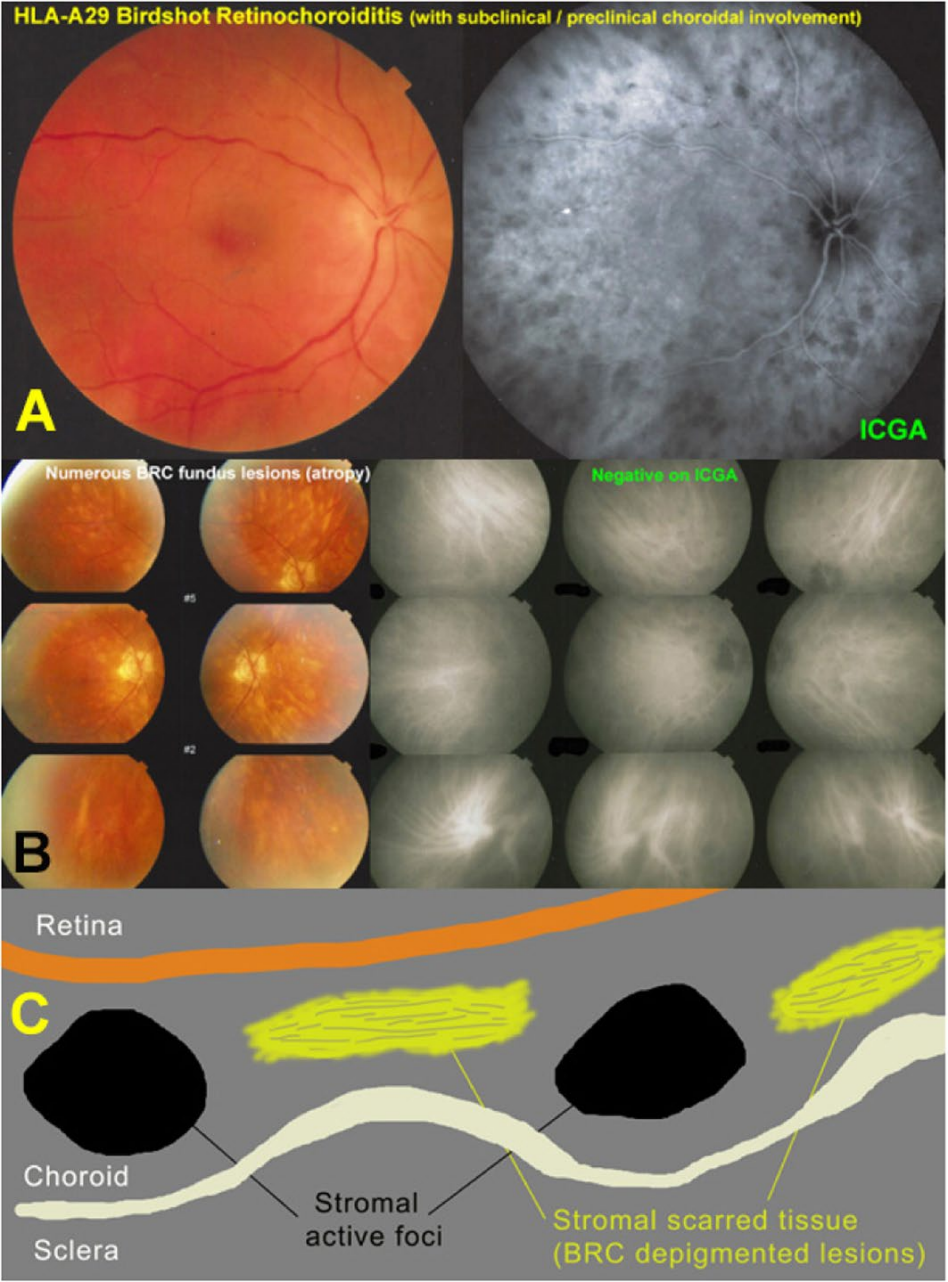
HLA-A29 BRC. ICGA is essential for early diagnosis of disease and for monitoring disease activity. **A** Patient complaining of floaters and “dim” vision. Because of bilateral oedematous optic discs, an angiography was performed showing numerous HDDs on ICGA in the posterior pole and midperiphery (top right). HLA-A29 testing was positive. **B** BRC patient who had been treated with immunosuppressives, with numerous cream-coloured BRC fundus lesions, who’s ICGA did not show any HDDs, indicating inactivity of disease. **C** cartoon inspired from Mirò, showing that cream coloured BRC lesions are choroidal scars (yellow discs), (not appearing on ICGA), while black round spheres represent active choroidal foci identified by ICGA. Fundus lesions and HDDs on ICGA should not be confused

BRC is a preoccupying diagnosis when it is announced to the patient because of the genetic predisposition it bears with it. Patients are often concerned for the other members of their family. Several of our patients asked us to search for the presence of the HLA-A29 antigen in their kinship. When HLA-A29 is negative by PCR or DNA sequencing methods in the relative, there is a close to 100% chance that the diagnosis can be excluded [4]**.** There is quasi no such a thing as an HLA-A29 negative BRC. HLA-A29 typing remains a "sine qua non" diagnostic criterion. In spite of recent classification criteria based on claims from case series contesting this idea, still speaking of a 90 to 95% positivity rate probably relying on outdated testing methods [5]**,** two genetic studies of over hundred patients indicate ~ 100% positivity for HLA-A29 [3, 6] Hence, it was necessary to rectify the inappropriate diagnostic criteria of 2006 [7], asserting that HLA-A29 was an essential diagnostic criterion and not only a “supportive” element, among other rectifications [8]**.** Indeed, the 32 BRC patients seen in our centre were all HLA-A29 positive [9]**,** as well as the patients included in a French cohort by Gelfman et al., showing that all 262 patients with birdshot were retrospectively HLA-A29 positive [6]**.**

Confronted with a BRC patient who fears that his relative may present the disease, in case of absence of HLA-A29, the answer of the clinician hence is quite simple and allows to reassure totally the family indicating that there is no chance to develop the disease. The situation is completely different when the relative is also HLA-A29 positive, a situation we encountered recently where the younger sister of a BRC patient was also HLA-A29 positive. Although HLA-A29 positivity is not sufficient to develop the disease and co-factors are needed to be present, it is not possible to assure with certainty that the disease will never develop. Indeed, familial cases of BRC have been described [10]. We asked ourselves how the situation should be handled practically? The patient was aware that other genes had been identified for BRC. Indeed, dysfunction at the level of ERAP1 and ERAP2 may favour BRC in HLA-A29-positive individuals [2, 3]**.** The absolute risk of BRC is significantly higher when all three risk gene variants are carried, but pre-emptive genetic testing for *ERAP1* and *ERAP2* polymorphisms does not improve BRC prediction in HLA-A29-positive individuals to a clinically meaningful extent [2, 6]**.**

Instead, it would be far more valuable to opt for pioneering pragmatism and have a clinical work-up performed and inform the healthy person of the possible symptoms. In case of such a preventive follow-up the crucial investigation to perform is ICGA, that can reveal subclinical/preclinical involvement through detection of occult hypofluorescent dark dots (HDDs) [11]** (**Fig. 2**).**

A complete uveitis work up was negative with normal visual fields and a normal ICGA in the relative of our patient. Because of the fears of the younger sister of our patient, we proposed an annual follow-up visit with visual field testing, one of the sensitive functional signs to monitor in BRC, and an ICGA in case of a suspicion. Until further genetic testing is routinely available that can potentially predict risk factors, the most sensible attitude in case of suspicion in a healthy HLA-A29 positive relative of a BRC patient, probably is to perform a relevant clinical examination that must include ICGA, the most sensitive modality to detect pre-clinical disease [11].

## Data Availability

For data, please refer to corresponding author.

## Abbreviations

HLA: Human leukocyte antigen
BRC: Birdshot retinochoroiditis
ICGA: Indocyanine green angiography
MCH-I: Major histocompatibility complex class I
ERAP: Endoplasmic reticulum aminopeptidase
PCR: Polymerase chain reaction
DNA: Deoxyribonucleic acid
HDDs: Hypofluorescent dark dots
